# A Review of Mr. Burns' Administration

**Published:** 1914-02-21

**Authors:** 


### The Hospital

The Workers' Newspaper of Administrative Medicine and Institutional Life, Administration, National Insurance and Health.

No. 1443, Vol. LV. SATURDAY, FEBRUARY 21, 1914. PRICE ONE PENNY

### A REVIEW OF MR. BURNS' ADMINISTRATION.

The announcement of Mr. Burns' appointment to the Presidency of the Board of Trade leads one naturally to review the changes he has inaugurated during the time he has had control of the Local Government Board—whatever views may be held as to his success all will agree that his work has been characterised by thoroughness and immense industry. He was appointed in 1905, at a time when the Poor-Law branch of the Board's work was the subject of severe criticism. The same year saw the appointment of the Royal Commission to Inquire into the Poor Laws and the Relief of Distress. It is interesting to look back and to reflect how completely the hopes and fears expressed when he took office have been falsified. Ardent Poor-Law and public health reformers hailed his advent as that of one who would introduce far-reaching changes. Many old officials and others who realised how much excellent work was being quietly carried out, and the complexity of the problems dealt with, looked forward with dread to the results of the iconoclastic zeal with which he was credited. To-day Mr. Burns' bitterest enemies are to be found amongst the reformers, his staunchest advocates amongst the more conservative of the members of the local governing bodies and their officials. The record of his work during the past nine years shows steady progress and great improvement in the methods and details of administration, but no great or revolutionary changes.

He has been responsible for many minor legislative Acts, but only one of them the Housing and Town Planning Act of 1909—is likely to have any far-reaching effect upon the life of the community. This Act made it compulsory upon all county councils to appoint a medical officer of health upon conditions to be prescribed by the Local Government Board as regards his duties and his tenure of office. It gave the Board powers to enforce the carrying out of the various Acts relating to the housing of the working classes, and to regulate the procedure to be adopted with respect to the preparation or adoption of town-planning schemes. The other Acts he has introduced and passed deal with minor matters of public health and local government. Only two of them are of sufficient importance to be noticed here—the Notification of Births Act, 1907, enabling and, under certain circumstances, requiring local authorities to adopt a system of compulsory notification of births, and the Public Health (Regulations as to Food) Act, 1907, authorising measures to be taken for the prevention of danger arising to public health from the importation, preparation, storage, and distribution of articles of food or drink intended for sale for human consumption.

Turning to the Poor-Law side of his work, the chief reforms he has undertaken have lain in the direction of codifying and simplifying the regulations made by his predecessors. Soon after his appointment the scandals in the administration of the Poor Law in West Ham and Poplar became the subject of public criticism. He dealt with them and minor scandals sternly, promptly, and efficiently, and the action he took then and later has done immense good in raising the level of Poor-Law administration throughout the country. In this connection reference must be made to the good effects of his refusal to sanction the appointment of members of Boards of Guardians to offices as paid officials, and to the excellent changes made in regard to casual wards within the Metropolis.

Throughout his term of office he has worked steadily, if more deliberately than many hoped, towards the improvement of the position of the children and the sick under the Poor Law. The policy of removing the children from the ordinary wards of the workhouse begun under his predecessors has been steadily pushed forward by him, with the result that whereas in 1908 4.7 per cent. of the total number of children over three in receipt of indoor relief were to be found in these wards the percentage had sunk in 1913 to 3.3. Under the Poor Law Institutions Order just issued the retention of children over three in the ordinary wards of the workhouse is forbidden after March 31, 1915. In 1911 a Boarding-out Order was issued which consolidated previous Orders on the subject and introduced some useful amendments. As regards the sick there has been a notable increase in the provision of accommodation for them separate from the workhouses and an improvement in the equipment of the wards and in the numerical strength of the nursing staff. Though very much still requires to be done, the condition of the sick under the Poor Law to-day is much better than it was at the time of the Report of the Royal Commission. As regards the mixed workhouse and the poor in receipt of out-relief the reforms introduced have been chiefly in matters of detail and in the introduction of uniform methods of administration for the whole country. The most important instance is the Poor Law Institutions Order, 1913, just issued, the provisions of which, as regards the medical officers, we dealt with fully last week. The Relief Regulation Order, 1911, introduced no new principles, but it substituted one uniform code for the previously existing regulations, and it made the use of the case paper system compulsory in all parishes. In London a beginning has been made in the direction of the provision of special accommodation for special classes dealt with under the Poor Law.

In Birmingham a still more important innovation has been sanctioned. Here the Union has been enlarged, and all its medical activities have been co-ordinated under a principal medical officer. The infirmaries have been classified and arrangements made for fully equipped $x$-ray and pathological departments. The most important of the other changes introduced by Mr. Burns are the Tuberculosis Regulations of 1908, 1911, and 1912 and the Vaccination Act of 1907. Under the tuberculosis regulations the notification of cases of pulmonary phthisis was made compulsory on Poor-Law medical officers as regards persons in receipt of relief in 1908; this was extended in 1911 to the medical officers of hospitals and dispensaries as regards patients of those institutions; and in 1912 the regulations were further extended to non-pulmonary forms of the disease. The Vaccination Act of 1907 is, perhaps, the only piece of legislation due to Mr. Burns open to severe condemnation from a public health point of view. By extending the facilities for obtaining exemption from vaccination it has led to a most serious decline in the protection of the community against the risk of outbreaks of small-pox.

